# Impact of Adjuvant Treatment on Heparanase Concentration in Invasive, Unilateral Breast Cancer Patients: Results of a Prospective Single-Centre Cohort Study

**DOI:** 10.3390/jcm10102184

**Published:** 2021-05-18

**Authors:** Barbara Ruszkowska-Ciastek, Kornel Bielawski, Elżbieta Zarychta, Piotr Rhone

**Affiliations:** 1 Department of Pathophysiology, Faculty of Pharmacy, Nicolaus Copernicus University, Collegium Medicum, 85-094 Bydgoszcz, Poland; kornel@doktorant.umk.pl (K.B.); or zarychta@abs.umk.pl (E.Z.); 2 Clinical Ward of Breast Cancer and Reconstructive Surgery, Oncology Centre Prof. F. Łukaszczyk Memorial Hospital, 85-796 Bydgoszcz, Poland; rhonep@co.bydgoszcz.pl or

**Keywords:** adjuvant therapy, heparanase, breast cancer, disease recurrence

## Abstract

Background: In recent years, great progress has been made in the treatment of breast cancer, but it is still one of the ten leading causes of death in women. The aim of the study was to evaluate the heparanase concentration of invasive breast cancer (IBrC) patients, before and after cancer adjuvant treatment. Methods: Eighty patients with stage IA to IIB IBrC receiving adjuvant treatment were included prospectively in this study. The heparanase concentrations were determined by an enzyme-linked immunosorbent assay. A univariate analysis was used to estimate the factors influencing the low or high pre-treatment concentration of heparanase and the low or high numerical decrease in heparanase concentration after completion of adjuvant treatment. Results: Treatment reduced the concentration of heparanase by almost four times in the general IBrC cohort. Higher levels of pre- and post-treatment heparanase were noted in oestrogen receptor-negative cancers than in positive ones. A higher post-treatment concentration of heparanase was found in patients with a triple-negative tumour compared to patients with a luminal B HER2 negative type of IBrC. Overweight IBrC subjects and those with a tumour diameter of ≥2 cm demonstrated a lower chance of a lower pre-treatment heparanase concentration. Interestingly, a pre-treatment heparanase concentration is the main predictor of the changes in heparanase concentration after adjuvant treatment. Follow-up revealed significantly lower progression-free survival (PFS) rates in IBrC patients with a pre-treatment concentration of heparanase higher than 181.46 pg/mL (PFS = 80%). Conclusions: Our findings provide supporting evidence that IBrC therapy reduced the heparanase levels, regardless of treatment patterns and a pre-treatment concentration of heparanase may serve as a prognostic indicator for future outcomes.

## 1. Introduction

Invasive breast cancer (IBrC) is one of the ten leading causes of death among women worldwide. According to data of the American Cancer Society, in the USA, about 13% of women in their lifetime will be diagnosed with IBrC, and every fifth patient will die due to this [1,2]. Therefore, early detection of a tumour is a fundamental component in IBrC therapy that ameliorates survival and the quality of the patient’s life. Due to the large diversity of IBrC phenotypes, the choice of an adjuvant therapeutic approach depends on molecular subtype, histological type, stage, menopausal status, and other co-morbidities [2,3].

Generally, there are two treatment strategies for IBrC: local, relying on surgery and radiotherapy; or systemic, composed of chemotherapy, endocrine therapy, or targeted therapies [4]. According to the American Cancer Society data, the most common surgical procedure among patients with stage I and II is breast-conserving surgery (BCS) with adjuvant radiotherapy, with similar survival rates to mastectomy [2]. Oestrogen receptor (ER) or progesterone receptor (PgR)-positive breast cancer is recognised in approximately 70–80% of IBrC patients [5]. These subjects can benefit from long-term endocrine treatment via a reduction in the risk of local and distant relapses. However, particular tumour profiles are essential in establishing the prognosis of patients with hormone receptor positive IBrC undergoing treatment with selective ER modulators (SERMs) and aromatase inhibitors (AIs) such as tamoxifen and anastrozol, respectively. Patients with luminal A-like tumours treated with tamoxifen have a better prognosis than those with luminal B-like tumours, since the latter shows a higher proliferation/mitotic index (Ki67) [5,6]. However, lack of receptor expression is associated with the most severe breast cancer phenotype, accounting for 10–20% of all IBrC, and called triple-negative IBrC. These patients present a higher relapse rate, are increasingly prone to forming brain or lung metastases, and have a reduced overall survival rate due to the failure of current targeted therapies [7]. Despite the introduction of precision medicine, there still is a high risk of short- and long-term mortality due to breast malignancy, thus new therapies are constantly being sought that will allow the greatest possible reduction in the number of relapses, deaths, or over-treatment of low-risk patients [8].

Uncontrolled proliferation, resistance to apoptosis, angiogenic potential, and motility are natural cancer cell attributes [9]. One of the first steps in tumour cell invasion and secondary spread is the breakdown of connections between cells and between cells and the extracellular matrix (ECM) at the primary site of the tumour. Local remodelling of the basement membrane (BM) also facilitates this process [10,11]. Heparanase is an endo-β(1,4)-D-glucuronidase able to hydrolyse heparan sulphate (HS) side chains into 5 to 7 kDa fragments found in the BM and ECM [4,12,13,14]. Interestingly, Hunter et al. have observed a reduction in HS levels at the invasive fronts of tumours [15]. Heparanase exerts its action via enzymatic and non-enzymatic ways. Interestingly, both heparanase activities are equally involved in cancer invasion and dissemination, allowing neoplastic cells to invade the tumour site locally and spread to distant sites [10,11]. By cleaving ECM proteins, heparanase can release and thus activate several latent growth factors including vascular endothelial growth factor (VEGF), basic fibroblast growth factor (bFGF), hepatocyte growth factor (HGF) attached at this site, which leads to the promotion of proliferation, migration, invasion, and cancer cell spread. Furthermore, the non-enzymatic activity of heparanase is associated with enhancement of the coagulation system and sets off a vicious circle [12,14] as heparanase is considered as a tissue factor (TF) cofactor; higher bioavailability of TF is associated with thrombin production and fibrin deposition. Thrombin activates platelets, which release heparanase from their α granules. TF directly influences the release of VEGF from endothelial cells [14]. Thus, heparanase directly leads to hypercoagulability and a neoangiogenic switch by upregulation of TF and VEGF. It is worth mentioning that heparanase exhibits a positive side through involvement in tissue regeneration and repair, wound healing, hair growth, dendritic cell migration, and the implantation of embryos during the early stages of pregnancy [12].

Heparanase over-secretion is observed in several solid tumours, sarcomas, haematological neoplasms, and is associated with aggressive tumour behaviour, a worse prognosis, and chemo-resistance [10,13]. According to our previous study, we suggest that patients considered low risk of relapse based on their pre-treatment heparanase values may better respond to adjuvant treatment [16]. In order to avoid distant metastasis, there is a growing need to discover novel non-invasive biomarkers for primary IBrC and a treatment response that allows the detection of changes in mammary glands at an early stage as well as potential therapy resistance. In the present study, we hypothesised that surgical procedures and standard adjuvant therapies influence the change in heparanase concentration of patients with unilateral, invasive breast cancer. We also evaluated whether pre- and post-treatment levels of heparanase are a valuable biomarker for assessing disease relapse and monitoring disease progression in patients with IBrC.

## 2. Materials and Methods

### 2.1. Study Design and Population

This was an adjuvant, observational, prospective, single-centre study including female patients with histologically proven resectable, unilateral, primary IBrC (M0). Since this was an observational study performed in a daily clinical practice setting, the sample size was dependent on obtaining patients’ consent for participation in the study and confirmation of the will to donate blood at least twice. This study was undertaken in accordance with the Declaration of Helsinki, following the guidelines and approval of the local Ethics Committee (permission number: KB 547/2015). Informed consent was obtained from all participants included in the study. Subjects with IBrC (*n* = 80) were under the care of medical staff from the Clinical Ward of Breast Cancer and Reconstructive Surgery, Oncology Centre in Bydgoszcz, Poland. The decision between BCS and mastectomy or sentinel lymph node biopsy and axillary lymph node dissection was determined according to institutional guidelines.

### 2.2. Recruitment Criteria

For all patients enrolled in the study, baseline characteristics, including demographic data and medical history were obtained during a medical interview. Female patients were eligible for inclusion in the study if they met the following criteria: (1) age 40 years or older, (2) primary, (3) unilateral, (4) lack of distant metastases, (5) stage IA to IIB invasive breast cancer, (6) full follow-up information. To minimise confounding with comorbid conditions that could influence heparanase concentration, patients with systemic disorders such as (1) cardiovascular disease, (2) hepatic, kidney failure, and endocrine diseases, (3) acute infections, (4) autoimmune disorders, (5) previous history of malignant disease, (6) bilateral invasive IBrC, (7) carcinoma in situ were excluded. Distant metastases were excluded by thoraco-abdomin-pelvic tomography and bone scintigraphy.

### 2.3. Tumour Characteristics

Tumour and nodal stage (7th edition of the American Joint Committee on Cancer (AJCC) TNM classification of malignant tumours) were derived from all the included patients. Expression of oestrogen receptor (ER), progesterone receptor (PgR), human epidermal growth factor receptor 2 (HER2), Ki67 were obtained immunohistochemically in order to stratify patients according to the molecular subtypes of IBrC. The molecular subtypes of IBrC included luminal A (ER+/PgR+/HER2−/Ki67 < 20%), luminal B HER2(-) (ER+/PgR+/−/HER2−/Ki67 ≥ 20%), luminal B HER2(+) (ER+ and/or PR+/HER2+/Ki67− all values), non-luminal HER2(+) (ER−/PR−/HER2+/Ki67− all values), and triple-negative (ER−/PR−/HER2−/Ki67− all values). Tumours were graded according to the Elston–Ellis grading system based on three components: (1) the amount of tubule formation, (2) the nuclear grade, and (3) the mitotic index, in order to stratify the breast cancer. Grade 1 with well-differentiated cells (low grade), grade 2 with moderately differentiated cells (intermediate grade), and grade 3 with poorly differentiated cells (high grade).

### 2.4. Therapeutic Procedures

Standard guidelines related to treatment patterns established by the National Comprehensive Cancer Network (NCCN) Guidelines for Practice were implemented in all patients. BCS was performed in 65 patients (81%), while 15 patients (19%) had a mastectomy. Thirty-eight subjects (47.5%) had adjuvant chemotherapy administered, applied in four to six cycles. Thirty cases received anthracycline-containing drugs and non-anthracycline-containing drugs were used in eight patients. The chemotherapy patterns and dosages depended on the institutional guidelines. A complete blood count and organ function test was performed before each chemotherapy cycle. In 68 patients (85%), endocrine therapy was administered. The type of treatment depended on menopausal status; 41 (51%) used tamoxifen (Egis Pharmaceuticals, Budapest, Hungary), 20% (*n* = 16) of these patients receiving aromatase inhibitors (AIs) (Arimidex (anastrozol), AstraZeneca, Cambridge, United Kingdom) combination of tamoxifen and AIs was used in seven patients (9%), but four patients (5%) were given another endocrine scheme, in an adjuvant setting. Twelve subjects did not receive endocrine therapy due to a small tumour diameter or a triple-negative (ER−/PR−/HER2−/Ki67−all values) subtype of IBrC. Radiotherapy was delivered according to institutional guidelines within 1–2 weeks after completion of the adjuvant chemotherapy. In the study group, a median dose of 45 gray (Gy) was delivered in 17–20 fractions over 4–6 weeks to the chest wall by applying tangential photon fields, and for subjects with N1 status, to the supraclavicular, infraclavicular, and axillary nodes using an anterior field matched to the tangential fields. Fifty-two (65%) breast-conserved patients received, in addition, a sequential boost of 10 Gy delivered in five fractions to the initial tumour bed using a direct electron field. Only 15 patients did not require adjuvant radiotherapy (20%). In this study, no patients received immunotherapy between the first and second blood collection. Erythropoietin or granulocyte monocyte colony-stimulating factor (GM-CSF) supplemented treatments were not administered.

### 2.5. Follow-Up and Survival Status

Cumulative survival was demonstrated by Kaplan–Meier graphs. During a median follow-up of 55.0 months (IQR 49–59 months), 11 events occurred including three (3.75%) loco-regional recurrences, two (2.5%) distant metastases, and six (7.5%) deaths. The recurrence rate was 13.75%. The fates of all included patients are regularly followed and collected from the moment the first blood sample was taken. The progression-free survival (PFS) was defined as the period between the enrolment date and the day of radiological evidence of disease relapse or cancer-related death, whichever occurred first.

### 2.6. Blood Samplings

Blood samples from all patients were collected twice in accordance with standard procedures. The first blood collection occurred 24 h before the surgical procedure (I—pre-treatment values). Collection of the second blood specimen (II—post-treatment values) took place a maximum of three months after the last cytotoxic infusion and generally eight months (IQR 6.2–10.7) after the tumour removal procedure to avoid the direct impacts of chemotherapy or surgical wound healing on the level of heparanase.

### 2.7. Biochemical Measurements

Pre-treatment and post-treatment blood samples were collected from a peripheral venipuncture by a fresh needle insertion procedure. This consisted of the following: Blood was drawn by venipuncture into BD Vacutainer^®^ Plus Plastic K_2_EDTA tubes (Becton Dickinson, Plymouth, UK) containing potassium ethylene-diaminetetraacetic acid in order to measure the concentration of heparanase. A commercially available enzyme-linked immunosorbent assay method was used to determine the amount of heparanase (Cloud-Clone Corp., Katy, TX, USA; catalogue number: SEA711Hu) in K_2_EDTA plasma, which was prepared by centrifugation at 3000 g at 4 °C for 15 min and then stored in aliquots at −80 °C until used. For all kits, the reaction mixture was added to a 96-well plate. Laboratory analysis was carried out blindly to clinical data.

The limit of heparanase detection was <12.1 pg/mL. The intra-assay coefficient of variation (within-run) was <10% with an inter-assay coefficient of variation (run-to-run) of < 12%. The subjects were separated as having low (*n* = 40) or high (*n* = 40) values, dichotomised using a cut-off for pre-treatment heparanase of 181.46 pg/mL, based on the median value for the whole study population. Additionally, based on the median concentration of post-treatment heparanase of 47.14 pg/mL, the patients were divided into two equal groups with low (*n* = 40) and high (*n* = 40) heparanase concentrations after tumour resection and completion of chemotherapy. The obtained results allowed the numerical and percentage decrease in heparanase concentration to be determined.

### 2.8. Immunohistochemical (IHC) Analysis of Molecular Determinants

Paraffin-embedded sections were prepared for immunohistochemistry staining (IHC) analysis of oestrogen receptor (ER), progesterone receptor (PgR), human epidermal growth factor receptor 2 (HER2), and expression of Ki67. Immunostained specimens were examined by a pathologist who was blind to the clinical data of the patients and scored according to the intensity of staining. ER or PgR status >1% were considered as positive. HER2 scores were estimated using the standard American Society of Clinical Oncology/College of American Pathologists guidelines reporting system on a scale of 0, 1+, 2+, and 3+. Tumours with 0 or 1+ scores were established as HER2-negative and those with 3+ scores were considered as HER2-positive. A 2+ score was recognised as equivocal and was tested for HER2 gene amplification by fluorescent in situ hybridisation techniques (FISH), in accordance with the manufacturer’s instructions. For the Ki67- proliferation index, we used a 15% threshold as the limit to define high/low proliferative cases [17]. Detailed procedures are published in our previous paper [16].

### 2.9. Statistical Methods

The distribution of variables was checked initially by the Shapiro-Wilks normality test. Data were expressed as medians and interquartile ranges (IQR, 25th–75th percentile). The Mann–Whitney and the ANOVA Kruskal–Wallis tests were used to compare non-dependent subgroups. Furthermore, the data were compared by means of a non-parametric Wilcoxon signed-rank test for two dependent variables. In order to determine the differences in the frequency of occurrence of selected features between patients with and without disease recurrence, multi-way tables along with Pearson’s χ2 values were provided. Progression-free survival (PFS) was calculated from the date of enrolment until the documented disease progression or cancer-related death. The association of pre- and post-treatment heparanase concentrations as risk factors with time-to-event endpoints were analysed with the log-rank test or the F Cox test and the Kaplan–Meier method was used to plot the corresponding PFS curves. The relationship between the plasma heparanase levels and clinical determinants of breast cancer was determined using a logistic regression method to obtain an odds ratio (OR) with 95% confidence intervals (CI). All performed analyses were summarised and reported in tables and figures. All *p*-values were two-tailed and a *p*-value < 0.05 was considered significant. All statistical analyses were performed using dedicated Statistica 13.1 software (StatSoft, Cracow, Poland).

## 3. Results

### Baseline Characteristics of the Study Subjects

The median observation period was 55 months (range 49–59 months). During follow-up, 11 events occurred (relapse rate 13.75%), including 6 deaths and 5 disease recurrences. A total of 80 treatment-naïve patients, with histologically documented, stage IA-IIB IBrC were enrolled in the study from November 2015 to June 2017. Patient characteristics are given in Table 1, Table 2 and Table 3. The median age was 54.5 years (interquartile range (IQR) 49–59 years). The median body mass index value was 26.3 kg/m^2^ (IQR 22.54–28.97 kg/m^2^). The tumours were small and moderate with a median size of 1.7 cm (range 0.4–3.5 cm). A total of 53 patients (66%) were postmenopausal. Thirty-nine patients had stage IA (49%), tumour grades 1 + 2 were confirmed in 64 cases (80%) and tumours were ER or PgR positive in 86% and 79%, respectively. 24% (*n* = 19) of the patients had metastasis to axillary lymph nodes. None of the patients had neoadjuvant treatment provided.

In the present study, we analysed the potential benefit of pre- and post-treatment heparanase concentration as a marker for breast tumour invasion, pro-angiogenic phenotype, treatment response, and prognosis. Adjuvant treatment reduced the concentration of heparanase in the general IBrC cohort, *p* < 0.0001, by almost four times. A similar decreasing effect was observed with regard to age, menopausal status, body mass index (Table 1).

Table 2 presents the impact of adjuvant treatment on heparanase concentrations in terms of selected parameters characterising the molecular nature of breast cancer. The adjuvant treatment led to a significant decrease in heparanase concentration with respect to molecular determinants. Interestingly, the pre- and post-treatment heparanase concentrations were significantly different with respect to hormonal receptor status. Higher levels of pre- and post-treatment heparanase in oestrogen receptor-negative cancers (*p* = 0.0279 and *p* = 0.0498, respectively) than in positive ones were noted. Additionally, a significantly higher pre-treatment concentration of heparanase was observed in progesterone receptor-negative cases (*p* = 0.0253).

Regardless of molecular type, tumour diameter, lymph node involvement, staging, and grade of tumour, the concentration of heparanase also significantly decreased after adjuvant treatment (Table 3). Depending on the tumour diameter, significantly higher pre- (*p* = 0.0281) and post-treatment (*p* = 0.0128) concentrations of heparanase were obtained in patients with T2 tumours (≥2 cm) with respect to T1 (<2 cm). A higher post-treatment concentration of heparanase was found in patients with a triple-negative tumour compared to patients with a luminal B HER2 negative type of IBrC (*p* = 0.0494); however, the pre-treatment heparanase level did not differ significantly between these groups.

The next step of the statistical analysis was to assess pre- and post-treatment heparanase concentrations depending on the type of introduced treatment (Table 4). Regardless of the applied therapy, the post-treatment concentrations of heparanase also decreased. It is worth noting that pre- (*p* = 0.0299) and post-treatment (*p* = 0.0321) concentrations of heparanase vary with respect to the type of endocrine therapy. The lowest pre-treatment concentration of heparanase was found in patients who will later qualify for tamoxifen therapy but the highest level was obtained among patients without endocrine therapy (this group consisted of patients with a triple-negative or small size IBrC). The lowest post-treatment concentration of heparanase was noted in the group of IBrC cases who received a combination of tamoxifen and inhibitor aromatase treatment.

**Figure 1:**
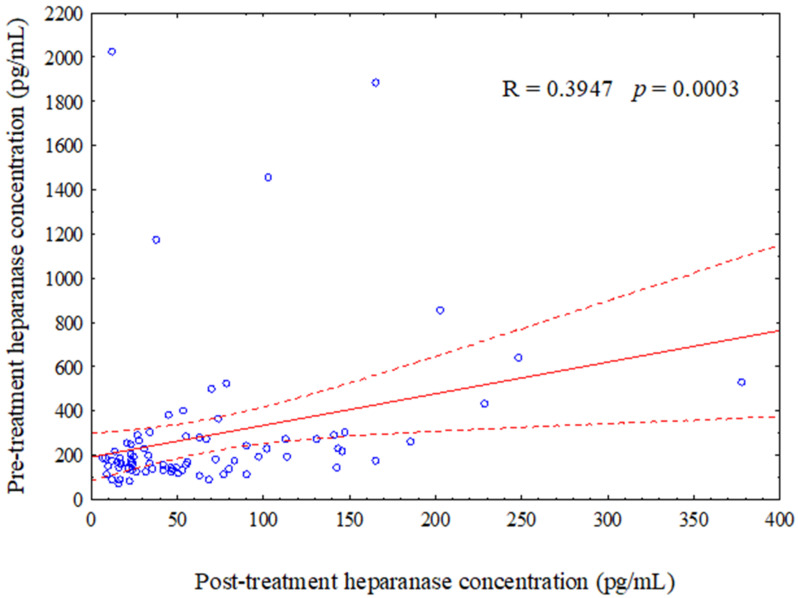
Scatter plot shows a positive of correlation between a pre-treatment with a post-treatment concentration of heparanase.

Figure 1 shows a significant positive correlation between a pre-treatment heparanase levels with post-treatment concentration of heparanase (*p* = 0.0003).

**Figure 2:**
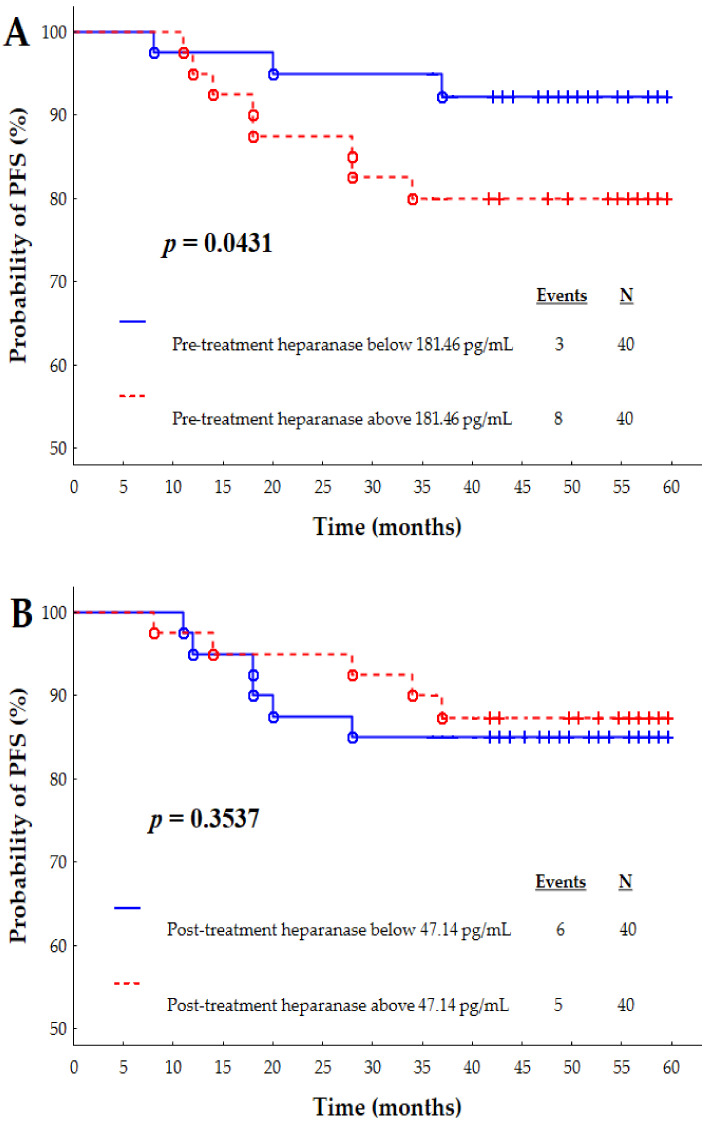
Kaplan-Meier survival curves depending on pre-treatment (**A**) and post-treatment (**B**) heparanase concentrations. The pre-treatment and post-treatment cut-offs for heparanase were set at 181.46 pg/mL and 47.14 pg/mL, respectively.

Interestingly, Figure 2A presents that the longer follow-up confirmed our previous findings [16] that a significantly higher incidence of disease relapse is observed in breast cancer patients with higher pre-treatment levels of heparanase compared to lower pre-treatment levels counterparts (log-rank *p* = 0.0431).

Figure 2B demonstrates the post-treatment evaluation of heparanase levels with respect to disease relapse. The probability of survival without recurrence of the neoplastic disease among patients with a post-treatment heparanase concentration higher than 47.14 pg/mL (12.5%) did not differ significantly (log-rank *p* = 0.3537) with regard to patients with a heparanase concentration lower than 47.14 pg/mL (15%).

**Figure 3:**
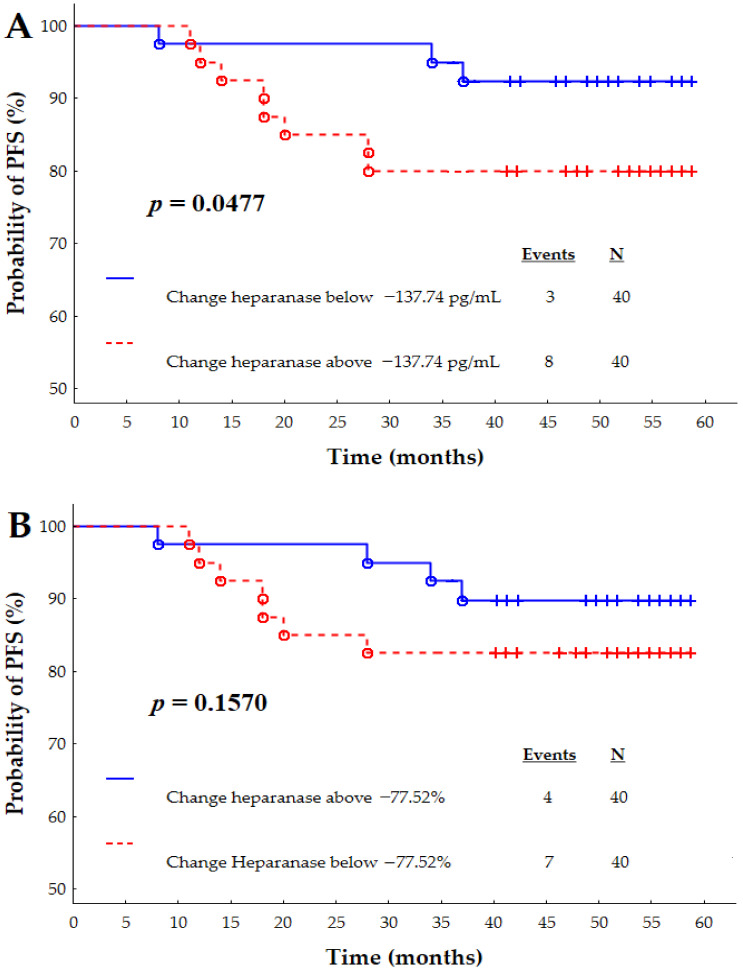
Kaplan-Meier survival curves depending on numerical (**A**) and percentage (**B**) changes of heparanase concentrations. The numerical and percentage changes cut-offs for heparanase were established at −137.74 pg/mL and −77.52%, respectively.

Additionally, IBrC patients were divided due to numerical and percentage changes in pre- and post-treatment heparanase concentrations (Figure 3A,B, respectively). Progression-free survival was lower in (log-rank *p* = 0.0477) in those patients with numerical changes in heparanase concentration above 137.74 pg/mL. Figure 3B showed that progression-free survival did not differ with regard to a low or high percentage change in pre- and post-treatment heparanase concentrations (log-rank *p* = 0.1570).

**Figure 4:**
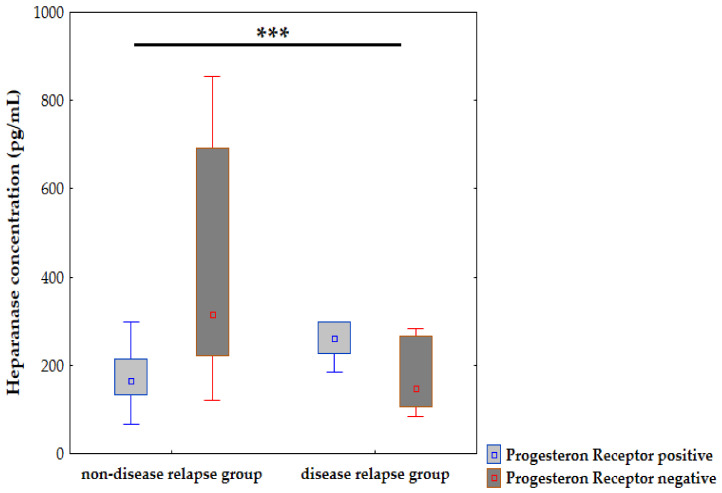
Progression-free survival depending on pre-treatment heparanase concentration and progesterone receptor status; significant difference is denoted by ***.

Figure 4 shows a significant difference in the pre-treatment concentration of heparanase (*p* = 0.0009) depending on progesterone receptor expression and disease relapse. Interestingly, patients with a positive PgR expression who developed a disease relapse showed a higher pre-treatment concentration of heparanase than in the patients without a disease relapse. However, among patients with a negative expression of PgR, disease recurrence occurred in those with a lower pre-treatment concentration of heparanase. This may indicate that a high pre-treatment concentration of heparanase may be a negative prognostic factor, thus other cancer-related factors should be taken into account.

The last step of the statistical analysis was to determine the factors influencing the low or high pre-treatment concentration of heparanase (Table 5) and the low or high numerical decrease in heparanase concentration after completion of adjuvant treatment (Table 6) using the univariate logistic regression.

On univariate analysis, overweight IBrC subjects and patients with a tumour diameter of ≥2 cm demonstrated a lower chance of a lower pre-treatment heparanase concentration (*p* = 0.0397, *p* = 0.0361, respectively). However, patients with a positive ER and PgR status showed a greater chance of having a lower pre-treatment heparanase concentration (*p* = 0.0369; *p* = 0.0191).

Table 6 demonstrates several factors which influence whether the change in pre- and post-treatment concentration of heparanase will be low or high. According to OR 0.97 and 95% CI 0.96–0.98; *p* < 0.0001 we suggest that the pre-treatment heparanase concentration is the main predictor of the changes in heparanase concentration after adjuvant treatment. In the subgroup of patients who received only tamoxifen compared to the subgroup of patients who did not receive any type of endocrine therapy, a small difference between the pre- and post-treatment heparanase levels was observed.

## 4. Discussion

Adjuvant treatment of IBrC depends not only on the tumour phenotype but also on the menopausal status and co-existing diseases. Thus, a wider perspective and more complex approach to IBrC treatment is crucial to better organising this process. In our study, we examined pre-treatment heparanase concentrations with respect to the adjuvant treatment response and we took efforts to determine the prognostic value of pre- and post-treatment heparanase levels and also numerical and percentage changes in pre- and post-treatment heparanase concentrations. Heparanase demonstrates strong implications for tumour aggressiveness and dissemination [13]. We compared the pre-treatment concentration of heparanase with its levels after surgery and adjuvant treatment. We observed that regardless of treatment pattern heparanase levels were reduced. Zhang et al. observed a significant decrease in serum heparanase concentrations in ovarian cancer patients after surgery [18]. It is worth mentioning that this observation is not obvious in all cancer types. Since, Ramani et al. observed that in eight of nine myeloma patients; tumour cells surviving chemotherapy had higher heparanase expression with respect to pre-treatment levels. The authors claim that in patients undergoing chemotherapy, NF-κB is stimulated leading to the upregulation of heparanase [19]. Bhattacharya et al. demonstrated that the activation and polarisation of macrophages induced by chemotherapy is also heparanase-linked, hence the widening of heparanase function in macrophages was noted [13]. Ramani et al. also demonstrated that a higher heparanase expression was associated with chemo-resistant cancer cells [20].

Tumour size, tumour stage, nuclear grade, and metastasis status have been reported to be of prognostic significance for IBrC. In the current study, we noted that the higher pre-treatment heparanase concentration is a good indicator for invasive phenotypes of breast cancer and shorter post-operative survival times. Furthermore, similar findings in respect to PFS were obtained for the numerical changes between first and second blood collection for heparanase. PFS was lower in those patients with a numerical change in heparanase concentration above 137.74 pg/mL.

Additionally, the pre-treatment heparanase concentration is the main predictor of the changes in heparanase concentration after adjuvant treatment. Interestingly, a positive correlation between pre-treatment heparanase levels with post-treatment concentration of heparanase confirms this hypothesis. This provides useful information that heparanase is a potent protein for cancer progression. Similarly, Vornicova et al. noted that high levels of heparanase in stage I breast cancer is linked with a 4.5-fold increased risk of disease recurrence [11]. Hunter et al. noted that heparanase mRNA was expressed at very low levels in normal islets, whereas its expression was increased 40-fold in primary tumours and metastatic tumours. Suppression of heparanase was associated with a significant decrease in tumour invasion [15]. Similarly, in normal breast epithelium, heparanase is undetected, but in breast malignancy, its expression elevates, which is linked with larger tumour size and aggressiveness [21]. Efficient heparanase activity depends on the microenvironment pH, since a pH of approximately 7 leads to inactivation of heparanase, but an acidic pH of between 5 and 6 provides its optimal activity during tumour growth and in pro-inflammatory conditions [22]. Zhang et al. established that either serum heparanase concentration or its expression at mRNA or/and protein levels indicate prognostic and diagnosis values in ovarian cancer [18]. Thus, overexpression of heparanase correlates positively with the invasion and spread of cancer cells as well as inflammation, procoagulant state and neovessel formation within the tumour. Since, heparanase is the only enzyme that degrades heparan sulphate, the main component of the ECM [14].

According to the univariate logistic regression analysis, we observed a lower chance of low pre-treatment heparanase levels in the overweight IBrC subjects compared to their normal-weight counterparts. Accumulation of adipose tissue is a well-known risk factor for hormone-dependent cancers. Interestingly, overweight/obesity in postmenopausal women is associated with increased susceptibility to luminal breast cancers, but premenopausal overweight/obese women are more predisposed to developing triple-negative cancer. Thus, adiposity is linked to more aggressive breast cancer behaviours, including more advanced tumour stage and poor survival [23,24]. Hyperinsulinemia is a causal link between adiposity and breast cancer development. Goldberg et al. demonstrated a strong interaction between heparanase and insulin signalling, which may support breast tumorigenesis. Since heparanase exerts properties to enhance insulin-induced proliferation in breast carcinoma cells in vitro, authors have reported a relevant association between lymph node metastases and the simultaneous existence of both hyperglycaemia and heparanase expression [25]. Thus, the reduced chance of having a lower pre-treatment concentration of heparanase in overweight IBrC cases most likely indicates worse future outcomes.

Analysing the heparanase concentration depending on the presence of oestrogen and progesterone receptors demonstrates higher pre-and post-treatment heparanase concentrations among ER(-) and PgR(-) patients compared to ER(+) and PgR(+) counterparts. The PFS rate was lower in PgR negative cases since the number of patients in this group was only 17 (PFS = 70.6%), while there were 63 patients with PgR positive expression, thus the PFS was 90.5% (Table S1). Additionally, according to univariate logistic regression, a tumour diameter larger than 2 cm, and the lack of ER and PgR expression were strong influences on high pre-treatment heparanase in IBrC patients. Our findings are in line with Imada et al.’s study, since the authors observed that the immunohistochemical identification of heparanase in breast cancer biopsy specimens was linked with a larger primary tumour size and tumour spread [26]. Tang et al. also observed that heparanase expression was positively connected with a larger size of breast cancer tumour, higher clinical stage, and lymph node metastasis [27,28]. Negative hormone receptor status with a higher concentration of heparanase is associated with a more aggressive nature of IBrC and a worse prognosis since the ER/PR negative subtypes of IBrC include a non-luminal HER2+ and a triple-negative molecular subtype. Both of these confer more aggressive character and clinical behaviour. These tumours the present higher histological and nuclear grades are highly proliferative and show poor tubule creation [29]. Thus, overexpression of heparanase leads to a loss of extracellular matrix integrity, enabling invasion, and the dissemination of cancer cells. Moreover, most likely higher post-treatment heparanase concentrations in ER/PR negative cancers was associated with chemo-resistant cancer cells and most likely with treatment failure [20]. However, Cohen et al. observed a three-fold incidence in the frequency of heparanase overexpression in ER-positive breast cancer with respect to the ER-negative ones. The authors claim that the ER-dependent heparanase regulation mechanism works in vivo, but the 15% occurrence of heparanase expression in ER-negative tumours suggests distinct molecular mechanisms are accountable for the increase in heparanase expression in the subgroup of ER-negative breast cancers [30]. Interestingly, according to our further analysis we observed that patients with a positive PgR expression who developed a disease relapse showed a higher pre-treatment concentration of heparanase than in the patients without a disease relapse. However, among patients with a negative expression of PgR, disease recurrence occurred in those with a lower pre-treatment concentration of heparanase. This may indicate that a high pre-treatment concentration of heparanase may be a negative prognostic factor, thus other cancer-related factors should be taken into account. Undoubtedly, further studies are needed in this field (Figure 4).

Finally, a higher post-treatment concentration of heparanase was found in patients with a triple-negative tumour compared to patients with a luminal B HER2 negative type of IBrC. According to our study (Table S1), regardless of tumour stage, triple-negative tumours (PFS = 67%) demonstrated more aggressive biological behaviour than tumours expressing HER2 (PFS = 100%). It is well-established that the triple-negative subtype of IBrC is identified by invasiveness and poor prognosis. The study of Yang et al. is in line with our findings that the expression of heparanase was significantly higher in the metastatic group with respect to their non-metastatic counterparts and a high expression of heparanase was also significantly linked with poor disease-free survival (DFS) and overall survival (OS) in triple-negative subjects. The authors have claimed that heparanase enhanced the blood and oxygen delivery of both the breast tumours and lung metastases through a new vessel network and vascular mimicry, thus leading to tumour growth and malignant progression [31].

Higher levels of heparanase may be provoked locally by its secretion from platelets, neutrophils, and mast cells [14]. Interestingly, tamoxifen can suppress tumour cell-induced platelet activation leading to a diminishing of the pro-angiogenic and pro-metastatic potential of platelets. A possible mechanism, by which this phenomenon may be explained, is based on platelets expressing oestrogen receptors α and β on their surface, but there are doubts whether tamoxifen influences platelets via these receptors, since tamoxifen is a selective oestrogen receptor agent that is generally used as anti-oestrogen therapy for breast cancer [32]. Our speculations related to reducing the release of heparanase from platelets by tamoxifen were based on Johnson’s study who noted that tamoxifen reduces the release of VEGF. The author suggests that tamoxifen ameliorates breast cancer survival rates by reducing the pro-angiogenic action of platelets [32]. Moreover, we observed that patients who received only tamoxifen had a smaller difference between the pre- and post-treatment heparanase levels than patients who did not receive any type of endocrine therapy. This could be due to two main reasons. Firstly, there is a link with the differences between the subgroups in pre-treatment heparanase concentrations, since the patients who did not receive endocrine therapy demonstrated negative oestrogen and progesterone receptor status as well as the highest pre-treatment heparanase level. However, with respect to pre-treatment concentrations of heparanase, the opposite dependencies were observed in the subgroup which was only given tamoxifen. Secondly, according to the analysis of the influence of an endocrine therapy scheme on the post-treatment heparanase concentration, the lowest heparanase concentrations were observed in patients who were first treated with tamoxifen and then with aromatase inhibitors. This may indicate the smaller effect of tamoxifen as a single agent on the reduction in heparanase concentration, and perhaps, therefore, in those subjects, we more often observed a low difference between pre- and post-treatment heparanase concentrations. Ekin et al. noted that oestrogen treatment stimulates heparanase gene transcription in oestrogen receptor-positive breast cancer cells in an in vitro study, leading to the development and progression of breast cancer [33]. Cohen et al. reported that tamoxifen demonstrates an oestrogen-like stimulatory impact on heparanase expression in two ER-positive breast carcinoma cell lines. The authors claim that most of the patients whose tumours were characterised by higher levels of heparanase were treated with tamoxifen [30].

### Limitations of the Study

One of the strengths of our study was its prospective nature, and also that the associations between the analysed factors and future outcomes were studied in a well-characterised study cohort and a long-term follow-up (median follow-up 55 months; IQR 49–59 months). The strength of this study was the incorporation of detailed clinicopathological data and wide ranges of potential prognostic indicators, which may have contributed to the favourable results. Some limitations of our study should be acknowledged. It was a single-centre study. The modest sample size precluded subgroup analyses (e.g., cancer histology types), limited multivariable analyses, and a limited assessment of confounding and interactions. The study population was fully Caucasian, limiting race/ethnicity association evaluations. Thus, these preliminary results need to be confirmed by a study on a large scale as well as by the functional analysis of heparanase through in vitro studies in the future to ensure the generalizability of our data. All in all, these limitations do not decrease the importance of our findings which can be easily implemented into clinical practice.

## 5. Conclusions

In conclusion, our findings indicate several relevant issues: (1) According to the expectations, IBrC therapy reduced the heparanase levels, regardless of treatment patterns; (2) Pre-treatment heparanase concentration is associated with the future outcomes of IBrC patients, since a concentration of heparanase higher than 181.46 pg/mL has been shown to promote the probability of recurrence and morbi-mortality in the IBrC cohort; (3) Interestingly, higher pre-treatment concentration of heparanase depends on tumour size (≥2 cm) and lack of ER and PgR expression; confirming its association with a more aggressiveness phenotype of the IBrC. Nevertheless, future studies should confirm the application of our cut-off point for the pre-treatment concentration of heparanase.

## Figures and Tables

**Table 1:** The impact of adjuvant treatment on the heparanase concentration with respect to age, menopausal status, body mass index (BMI).

Feature/ Number of Patients (%)	Pre-Treatment Heparanase Concentration (pg/mL)	Post-Treatment Heparanase Concentration (pg/mL)	*p*-Values
80 (100%)	181.46 138.86/269.60	47.14 23.20/86.92	<0.0001
Age	*p* = 0.8595	*p* = 0.2982	
<55 years 40 (50%)	179.23 133.57/280.18	43.78 17.61/74.01	<0.0001
≥55 years 40 (50%)	182.59 142.06/256.81	50.38 24.23/94.06	<0.0001
Menopausal status	*p* = 0.2396	*p* = 0.3159	
Premenopausal 27 (34%)	165.88 123.09/257.96	42.06 16.91/77.55	<0.0001
Postmenopausal 53 (66%)	188.24 144.19/270.91	47.72 24.18/90.43	<0.0001
BMI	*p* = 0.2020	*p* = 0.9578	
≤24.9 kg/m^2^ 38 (47%)	170.30 134.32/252.87	43.78 24.18/77.55	<0.0001
25–29.9 kg/m^2^ 27 (34%)	213.74 150.63/286.91	53.31 23.2/97.68	<0.0001
30–39.9 kg/m^2^ 15 (19%)	157.13 137.80/257.96	47.02 18.00/143.10	0.0003

BMI: body mass index.

**Table 2:** The heparanase concentrations according to molecular characteristics in breast cancer subjects.

Feature/ Number of Patients (%)	Pre-Treatment Heparanase Concentration (pg/mL)	Post-Treatment Heparanase Concentration (pg/mL)	*p*-Values
Expression of Ki67	*p* = 0.0967	*p* = 0.5052	
< 15% 42 (53%)	168.09 134.32/213.74	48.72 24.08/90.43	<0.0001
≥ 15% 38 (47%)	225.68 142.05/297.89	43.78 22.71/78.88	<0.0001
Expression of HER2	*p* = 1.0000	*p* = 0.9436	
Negative 72 (90%)	183.71 138.86/268.29	47.37 23.20/86.92	<0.0001
Positive 8 (10%)	164.81 131.53/390.19	46.38 21.87/109.70	0.0133
ER status	*p* = 0.0279	*p* = 0.0498	
Positive 69 (86%)	170.30 135.68/257.96	41.95 22.71/78.88	<0.0001
Negative 11 (14%)	268.29 213.74/378.49	73.88 45.50/146.50	0.0026
PgR status	*p* = 0.0253	*p* = 0.1595	
Positive 63 (79%)	170.30 135.68/252.87	42.06 22.23/80.49	<0.0001
Negative 17 (21%)	265.69 197.40/378.49	63.43 28.41/143.40	0.0001

Ki67: proliferation marker; HER2: human epidermal growth factor receptor 2; ER: oestrogen receptor; PgR: progesterone receptor.

**Table 3:** The heparanase concentrations with respect to clinical and pathological characteristics in breast cancer subjects.

Feature/ Number of Patients (%)	Pre-Treatment Heparanase Concentration (pg/mL)	Post-Treatment Heparanase Concentration (pg/mL)	*p*-Values
Molecular subtypes	*p* = 0.5157 II vs. IV 0.2756	*p* = 0.3135 II vs. IV 0.0494	
Luminal A 47 (59%)	165.88 134.32/257.96	47.02 22.23/90.29	<0.0001
Luminal B HER2(-) 16 (20%)	192.82 156.05/268.30	32.61 23.10/59.03	0.0002
Luminal B HER2(+) or non-Luminal HER2(+) 8 (10%)	164.81 131.53/390.19	46.38 21.87/109.70	0.0133
Triple-negative 9 (11%)	245.32 213.74/284.21	73.88 55.25/143.40	0.0077
Tumour diameter	*p* = 0.0281	*p* = 0.0128	
T1 (<2 cm) 53 (66%)	165.88 135.68/228.10	35.72 22.13/67.25	<0.0001
T2 (≥2 cm < 5 cm) 27 (34%)	257.96 157.13/297.89	70.47 34.42/131.40	<0.0001
Nodal status	*p* = 0.5224	*p* = 0.1277	
N0 61 (76%)	183.71 142.05/276.15	53.31 23.20/102.9	<0.0001
N1 19 (24%)	179.21 131.46/252.87	34.53 23.20/55.25	<0.0001
Tumour stage	*p* = 0.3676	*p* = 0.4549	
IA 39 (49%)	170.30 139.92/240.35	45.50 22.13/83.54	<0.0001
IIA + IIB 41 (51%)	197.40 137.80/270.91	51.03 24.68/90.29	<0.0001
Tumour grade	*p* = 0.9099	*p* = 0.7252	
G1 + G2 64 (80%)	176.98 140.99/269.60	47.49 22.72/86.92	<0.0001
G3 16 (20%)	219.71 131.49/274.95	43.73 25.81/111.95	0.0002

HER2: human epidermal growth factor receptor 2.

**Table 4:** The heparanase concentrations according to the types of surgery and adjuvant therapy in IBrC subjects.

Feature/ Number of Patients (%)	Pre-Treatment Heparanase Concentration (pg/mL)	Post-Treatment Heparanase Concentration (pg/mL)	*p*-Values
BCS + Radiotherapy- BCT 65 (81%) Mastectomy 15 (19%)	*p* = 0.9029 183.71 139.92/276.15 170.30 137.80/257.96	*p* = 0.9029 47.25 22.71/83.54 38.47 28.41/90.29	<0.0001 0.0003
Chemotherapy	*p* = 0.6731	*p* = 0.8818	
Anthracycline 30 (37.5%)	200.99 131.46/297.89	46.38 26.83/72.81	<0.0001
Non-anthracycline 8 (10%)	170.30 143.14/219.71	48.69 18.49/91.30	0.0003
No 42 (52.5%)	179.23 139.92/257.96	47.37 22.13/90.43	<0.0001
Endocrine therapy	*p* = 0.0299	*p* = 0.0321	
Tamoxifen 41 (51%)	150.63 131.46/225.68	42.06 24.08/80.49	<0.0001
Inhibitor aromatase 16 (20%)	191.66 174.76/277.60	42.78 24.09/105.74	0.0002
Tamoxifen and inhibitor aromatase 7 (9%)	183.71 154.96/286.91	16.91 12.73/47.02	0.0233
Other type 4 (5%)	174.80 124.13/229.93	39.65 19.96/59.76	0.1336
No 12 (15%)	276.25 220.92/454.46	102.64 50.38/156.05	0.0015

BCS- breast-conserving surgery; BCT- breast-conserving therapy.

**Table 5:** Univariate logistic regression analyses of low or high pre-treatment heparanase concentration predictors in IBrC patients.

Variable Code		Univariable	
		OR (95% CI)	*p*-Values
Age	<55 years	Reference	
	≥55 years	1.00 (0.42–2.40)	1.0000
Menopausal status	Premenopausal	Reference	
	Postmenopausal	0.71 (0.28–1.81)	0.4788
BMI	≤24.9 kg/m^2^	Reference	
	25–29.9 kg/m^2^	0.36 (0.13–1.02)	0.0397
	30–39.9 kg/m^2^	1.09 (0.32–3.69)	0.3138
Expression of HER2	Negative	Reference	
	Positive	1.76 (0.39–7.93)	0.4605
Expression of Ki67	< 15%	Reference	
	≥ 15%	0.55 (0.22–1.33)	0.1808
ER status	Negative	Reference	
	Positive	5.52 (1.11–27.43)	0.0369
PgR status	Negative	Reference	
	Positive	4.33 (1.27–14.78)	0.0191
Molecular subtypes	Luminal A	Reference	
	Luminal B HER2(-)	1.35 (0.29–6.30)	0.2404
	Luminal B HER2(+) or non-Luminal HER2(+)	0.63 (0.20–1.97)	0.9047
	Triple negative	0.23 (0.04–1.23)	0.1011
Tumour diameter	T1 (<2 cm)	Reference	
	T2 (≥2 cm <5 cm)	0.35 (0.13–0.94)	0.0361
Nodal status	N0	Reference	
	N1	1.15 (0.41–3.22)	0.7928
Tumour stage	IA	Reference	
	IIA + IIB	0.49 (0.20–1.20)	0.1193
Tumour grade	G1 + G2	Reference	
	G3	0.73 (0.24–2.20)	0.5769

OR: odd ratio; CI: confidence interval; BMI: body mass index; Ki67: proliferation marker; HER2: human epidermal growth factor receptor 2; PgR: progesterone receptor.

**Table 6:** Univariate logistic regression analyses of low or high heparanase changes as predictors in breast cancer patients.

Variable Code		Univariable	
		OR (95% CI)	*p*-Values
Age	<55 years	Reference	
	≥55 years	1.22 (0.51–2.94)	0.6549
Menopausal status	Premenopausal	Reference	
	Postmenopausal	0.89 (0.35–2.26)	0.8131
BMI	≤24.9 kg/m^2^	Reference	
	25–29.9 kg/m^2^	0.53 (0.19–1.45)	0.0683
	30–39.9 kg/m^2^	1.80 (0.52–6.27)	0.1344
Expression of HER2	Negative	Reference	
	Positive	1.00 (0.23–4.31)	1.0000
Expression of Ki67	<15%	Reference	
	≥15%	0.44 (0.18–1.09)	0.0753
ER status	Negative	Reference	
	Positive	1.91 (0.51–7.12)	0.3356
PgR status	Negative	Reference	
	Positive	2.15 (0.71–6.53)	0.1771
Molecular subtypes	Luminal A	Reference	
	Luminal B HER2-	0.74 (0.17–3.33)	0.7579
	Luminal B HER2+ or non-Luminal HER2+	0.34 (0.10–1.12)	0.2014
	Triple-negative	0.59 (0.14–2.49)	0.9350
Tumour diameter	T1 (<2 cm)	Reference	
	T2 (≥2 cm <5 cm)	0.89 (0.35–2.26)	0.8131
Nodal status	N0	Reference	
	N1	0.87 (0.31–2.44)	0.7928
Tumour stage	IA	Reference	
	IIA + IIB	0.90 (0.38–2.17)	0.8230
Tumour grade	G1 + G2	Reference	
	G3	1.00 (0.33–2.99)	1.0000
Pre-treatment heparanase concentration (pg/mL)	Continuous	0.97 (0.96–0.98)	<0.0001
Post-treatment heparanase concentration (pg/mL)	Continuous	1.00 (0.99–1.01)	0.7053
Surgery type	BCS	Reference	
	Mastectomy	1.18 (0.38–3.63)	0.7747
Endocrine therapy	No	Reference	
	Tamoxifen	3.47 (0.89–13.48)	0.0313
	Inhibitor aromatase	1.20 (0.25–5.77)	0.6984
	Tamoxifen and inhibitor aromatase	0.80 (0.10–6.10)	0.4027
	Other	2.00 (0.20–19.91)	0.7070
Chemotherapy	No	Reference	
	Anthracycline	1.00 (0.39–2.55)	1.0000
	Non-anthracycline	1.00 (0.22–4.54)	1.0000
Radiotherapy	No	Reference	
	Yes	0.73 (0.24–2.20)	0.5770

OR: odd ratio; CI: confidence interval; BMI: body mass index; Ki67: proliferation marker; HER2: human epidermal growth factor receptor 2; PgR: progesterone receptor; BCS- Breast-conserving therapy.

## Data Availability

The data presented in this study are available in this article and supplementary material.

## Supplementary Materials

The following are available online at https://www.mdpi.com/article/10.3390/jcm10102184/s1. Table S1: Informative profile of IBrC patients in respect to disease relapse.
