# Supplementary material for: Impact of Adjuvant Treatment on Heparanase Concentration in Invasive, Unilateral Breast Cancer Patients: Results of a Prospective Single-Centre Cohort Study
Source: J Clin Med. 2021 May 18;10(10):2184. doi: 10.3390/jcm10102184 (PMC8158114; doi:10.3390/jcm10102184)
Supplement: Supplementary file 1 [file jcm-10-02184-s001.zip › jcm-1213881-SI.pdf]

Supplementary Materials

Table S1 demonstrates the factors that occurred significantly more frequently among patients with disease recurrence. Progression-free survival rates were lower in patients with a BMI ≤ 24.9 kg/m^2, expression of Ki67 > 15 % and stage IIA-IIB, since nine out of 11 cases with disease relapse (PFS = 76.3 %, 76.3 %, 78 %, respectively) had a BMI ≤ 24.9 kg/m^2, expression of Ki67 > 15 % and stage IIA-IIB. According to the analysis of disease relapses depending on progesterone receptor expression (positive or negative), similar numbers of disease relapses were obtained in both subgroups (six and five events, respectively). However, the PFS rate was lower in PgR negative cases since the number of patients in this group was only 17 (PFS = 70.6 %), while there were 63 patients with PgR positive expression, thus the PFS was 90.5 %. Similar differences occurred in the subgroups formed with respect to tumour diameter. In the subgroup with a tumour diameter < 2 cm, recurrent events happened in three patients of the total of 53 followed-up subjects (5.7 %), whereas in the subgroup with a tumour diameter ≥ 2 cm, recurrent events occurred in eight cases in the total of 27 followed-up subjects (29.6 %). The incidence of disease relapses also varies with the molecular subtype of IBrC. In the worst-prognostic subgroup were patients with a triple-negative IBrC, since there was a disease relapse in three patients out of a total of nine (disease relapse rate was 33 %). Luminal A HER(-) subtype demonstrates similar lower PFS rate (31 %), since five out of the 16 cases had disease relapse. The luminal A and luminal B HER2(+) and non-luminal HER2(+) subtypes demonstrate the longest progression-free survival compared to the other molecular subtypes. The incidence of disease relapse in cases with luminal A IBrC was three out of 47 patients (PFS = 93.6 %), while none of the eight patients with luminal B HER2(+) and non-luminal HER2(+) had disease recurrence (PFS = 100 %).

Table S1. Informative profile of IBrC patients in respect to disease relapse

Feature		Disease relapse Yes	Disease relapse No	Chi^2	*P*-values
**BMI**	≤ 24.9 kg/m^2	9 (11%)	29 (36%)	6.09	**0.0475**
	25-29.9 kg/m^2	1 (1%)	26 (33%)		
	30-39.9 kg/m^2	1 (1%)	14 (18%)		
Expression of Ki67	< 15%	2 (3%)	40 (50%)	6.02	**0.0141**
	≥ 15%	9 (11%)	29 (36%)		
PgR status	Negative	5 (6%)	12 (15%)	4.46	**0.0346**
	Positive	6 (8%)	57 (71%)		
Tumour diameter	T1 (<2 cm)	3 (4%)	50 (63%)	8.67	**0.0032**
	T2 (≥2cm< 5cm)	8 (10%)	19 (24%)		
Tumour stage	IA	2 (3%)	37 (46%)	4.77	**0.0290**
	IIA+IIB	9 (11%)	32 (40%)		
Molecular subtypes	Luminal A	3 (4%)	44 (55%)	10.47	**0.0150**
	Luminal B HER2(-)	5 (6%)	11 (14%)		
	Luminal B HER2(+) or non-Luminal HER2(+)	0 (0%)	8 (10%)		
	Triple-negative	3 (4%)	6 (8%)		

BMI: body mass index; Ki67: proliferation marker; PgR: progesterone receptor; significant differences are denoted by bold.
